# Paricalcitol versus Calcitriol + Cinacalcet for the Treatment of Secondary Hyperparathyroidism in Chronic Kidney Disease in China: A Cost-Effectiveness Analysis

**DOI:** 10.3389/fpubh.2021.712027

**Published:** 2021-07-21

**Authors:** Zhuolin Zhang, Lele Cai, Hong Wu, Xinglu Xu, Wenqing Fang, Xuan He, Xiao Wang, Xin Li

**Affiliations:** ^1^Department of Clinical Pharmacy, School of Pharmacy, Nanjing Medical University, Nanjing, China; ^2^Department of Pharmacy, Lianyungang Second People's Hospital, Lianyungang, China; ^3^Department of Health Policy, School of Health Policy and Management, Nanjing Medical University, Nanjing, China; ^4^Center for Global Health, School of Public Health, Nanjing Medical University, Nanjing, China

**Keywords:** paricalcitol, calcitriol, cinacalcet, secondary hyperparathyroidism, chronic kidney disease, cost-effectiveness analysis

## Abstract

**Background:** Chronic Kidney Disease (CKD) is a global chronic disease with increasing prevalence in recent years, particularly CKD accompanied by Secondary Hyperparathyroidism (SHPT) leads to reduced quality of life, increased mortality, a considerable economic burden for patients and society. The aim of this study was to investigate the cost-effectiveness analysis of paricalcitol vs. calcitriol + cinacalcet for CKD patients with SHPT in China in 2020.

**Methods:** A Markov model was conducted employing data derived from published literature, clinical trials, official sources, and tertiary public hospital data in China, based on a 10-year horizon from the perspective of the healthcare system. Calcitriol + Cinacalcet was used as the reference group. CKD stage 5 (CKD-5) dialysis patients suffering from SHPT were included in the study. Effectiveness was measured in quality-adjusted life years (QALYs). The discount rate (5%) was applied to costs and effectiveness. Sensitivity analysis was performed to confirm the robustness of the findings.

**Results:** The base case analysis demonstrated that Patients treated with paricalcitol could gain an increase in utility (0.183 QALYs) and require fewer expenditures (6925.612 yuan). One-way sensitivity analysis was performed to showed that impact factors were the price of cinacalcet, the hospitalization costs of patients with paricalcitol and calcitriol, the costs and utilities of hemodialysis and the costs of calcitriol, the costs of paricalcitol regardless of period. Probabilistic simulation analysis displayed when willingness-to-pay was ¥217113, the probability that Paricalcitol was dominant is 96.20%.

**Conclusion:** The results showed that paricalcitol administrated to treat patients diagnosed with Secondary hyperparathyroidism in Chronic Kidney Disease, compared to calcitriol and cinacalcet, might be dominant in China.

## Background

Chronic Kidney Disease (CKD) is a global chronic disease with gradually renal function loss. The number of individuals diagnosed with CKD and who die from CKD in China ranks top worldwide (1). The prevalence of CKD, even exceeding that of diabetes mellitus (2), has increased progressively from 10.8 to 11.6% during 2012–2018 in China (3, 4). The aging population deteriorates the prevalence of CKD in China, as well as a physiological decline in renal function and rising prevalence of Hypertension and Diabetes (3, 5). A cross-sectional survey conducted in China revealed that ~3 million CKD patients arrived in the end-stage renal disease (ESRD) stage over 3 years (4), where CKD patients are necessary to receive renal replacement treatment (RRT) (6). A large population of potential patients exists in China (4), as estimated, the prevalence of ESRD grown at a rate of 1.95% from 2015 to 2020 in Nanjing (7), a medium-sized city of China, imposing enormous pressure on the health care system due to productivity loss and premature death (8, 9).

Secondary hyperparathyroidism (SHPT) is a common serious complication in dialysis patients (10), over 70% advanced CKD patients are diagnosed with SHPT (11). As reported, about 60% of maintaining hemodialysis patients were affected by different levels of severity of SHPT, where the standard-reaching rate was about merely 55% (12). Due to renal dysfunction resulting in increased endogenous calcitriol and serum calcium, while decreased serum phosphorus (13), SHPT is characterized by increased serum parathyroid hormone (PTH) and parathyroid hyperplasia (14), risk factors of cardiovascular disease and fracture, resulting in lower quality of life, and higher mortality (10, 15, 16).

In 2017, total Medicare expenditure for CKD and ESRD in the US was high, approaching over 120 billion dollars (17). The same situation occurs in China in that the medical spending of CKD patients was 27.646 billion RMB in 2016, accounted for 6.50% of total health spending. For dialysis patients, the medical expenditure approached 911 million RMB, 75.6% of that was covered by urban basic health insurance. The economic burden of ESRD rises over time (9). It has been established that the medical expenditures of ESRD are multiplying at a mean annual rate of 5.8% in Nanjing (7). Treatment of CKD and ESRD is a heavy burden for patients, for family, and the health care system. Undoubtedly, Complications like SHPT, CVD exacerbate the economic burden for dialysis patients as well (18–20).

Based on the guideline-recommended by Kidney Disease: Improving Global Outcomes (KDIGO), Vitamin D Receptor Activators (VDRA) and calcimimetics are the optimal strategies of SHPT treatment, complemented to decrease PTH, prevent hypocalcemia, improve survival, and reduce cardiovascular disease (15, 21). Approximately 60% of patients undergoing hemodialysis and peritoneal dialysis were treated with Calcitriol, a non-selective Vitamin D (18). However, the adverse effects of Calcitriol due to non-selective mechanisms, hyperphosphatemia, and hypercalcemia causing vascular and soft tissue calcification (13)—the risk factors of mortality, restricts the effectiveness of calcitriol on the treatment of SHPT (10, 22, 23). Cinacalcet, the first and the only calcimimetics launched in China, approving for the treatment of maintaining dialysis (12). It has been documented that Cinacalcet has the advantage of quick onset in declining serum calcium, serum phosphorus, and PTH, meanwhile, reduce volume and size of parathyroid glands, hardly causing hyperphosphatemia and hypercalcemia (15, 24). Cinacalcet was commonly adopted when Vitamin D was poorly controlled (12).

Paricalcitol, a synthetic analog of vitamin D, less hypercalcemia and hypercalcemia, effectively decreases PTH, particularly administrated intravenously compared to the Calcitriol (15, 25, 26). As reported, in the long term, paricalcitol has a significant survival advantage and decreases hospitalization (4, 27–29). Notably, paricalcitol could be effective for calcitriol-resistant hemodialysis patients (23, 30).

Therefore, here we choose calcitriol and cinacalcet as references to explore the cost-effectiveness analysis of Paricalcitol. The cost-effectiveness of paricalcitol has been proved in Italian, US, UK, Brazil, German (13, 31–34). We aim to explore the cost-effectiveness of paricalcitol in China.

## Methods

### Model Design

**Figure 1:**
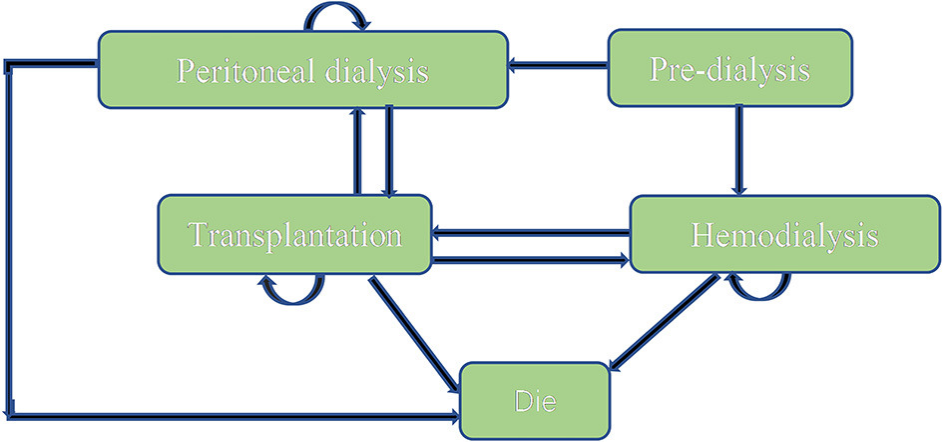
Markov model bubble diagram.

A Markov model (Figure 1) was conducted by Microsoft® Excel (Excel 2019 version) based on the model Nujiten (31) published. We invited local experts to confirm the Markov model is reasonable by requiring nephrology and pharmacoeconomics specialists' advice. The Markov model was selected for the following reasons: Markov model allows us to assess the cost-effectiveness of paricalcitol in the long term by defining a series of health states and health state transitions between these states to simulate disease process and combining consumable costs with health outcomes in every health states (35), moreover, it could take complications into considerations (31).

We assumed that a hypothetical cohort of 10,000 patients step into the model to calculate the standard error to make probabilistic sensitivity analysis and set that each cycle of the model was 1 year. All patients were in the “peritoneal dialysis” state (PD) or “hemodialysis” (HD) state at the beginning, the proportion distribution was extracted from the annual report on kidney in China (18). Patients could transfer to “Transplantation” state or “Dead” state, also could stay the original state after 1-year cycle. “Transplantation” and “peritoneal dialysis,” “Transplantation,” and “hemodialysis” were possible to convert each other. Importantly, “Dead” was an absorbed state and could not move back to other states. We hypothesized that the time horizon of 10 years since a study conducted in China (*n*= 153) reported that the survival rates from 5 years to 10 years for hemodialysis and peritoneal hemodialysis elderly patients dropped to 18.4 and 2.6% (36), which was appropriate because it covered the entire process from the moment they were diagnosed. The annual discount rate of costs and health outcomes was 5%.

### Cost Assessment

As shown in Table 1, the medical costs incorporated in this model included the costs of management of SHPT (paricalcitol, calcitriol, and cinacalcet), the costs of inpatient due to common complications (cardiovascular disease, fracture, and a series of complications), and the costs of management of ESRD (peritoneal dialysis, hemodialysis, transplantation) from the perspective of the health care system. The total cost per one patient was assessed once a year. All costs were presented and measured as Renminbi (RMB) in 2020.

**Table 1:** Inputs to model for base case analysis and sensitivity analysis.

**Costs (yuan/year)**	**Base case analysis**	**One-way sensitivity analysis**		**Distribution**	**References**
		**min**	**max**		
**Drug costs**					
Paricalcitol 1st year	21766.86	10448.09	26120.23	Gamma (α = 10000.00, β = 2.18)	(25, 37)
Paricalcitol beyond 1st year	18562.86	8910.17	22275.43	Gamma (α = 10000.00, β = 1.86)	(37)
Calcitriol	3942.00	1892.16	4730.40	Gamma (α = 10000.00, β = 0.39)	Official sources
Cinacalcet	27740.00	13315.20	33288.00	Gamma (α = 10000.00, β = 2.77)	(38)
**Renal replacement costs**					
Kidney transplantation 1st year	188697.90	150958.32	226437.48	Gamma (α = 560.06, β = 336.92)	Hospital data
Kidney transplantation beyond 1st year	99228.25	47629.56	119073.89	Gamma (α = 194.63, β = 509.83)	Hospital data
HD	67200.00	32256.00	80640.00	Gamma (α = 10000.00, β = 6.72)	(39)
PD	51600.00	24768.00	61920.00	Gamma (α = 10000.00, β = 5.16)	(39)
**Inpatient costs**					
Calcitriol + Cinacalcet	19375.57	15500.46	23250.68	Gamma (α = 58.26, β = 332.59)	Hospital data
Paricalcitol	19557.85	15646.28	23469.42	Gamma (α = 10.90, β = 1793.48)	Hospital data
**Utility (QALY/year)**					
HD	0.60	0.54	0.66	Beta (α = 1638.584, β = 1092.39)	(40)
PD	0.60	0.54	0.66	Beta (α = 1638.584, β = 1092.39)	(40)
Kidney transplantation	0.84	0.76	0.92	Beta (α = 7238.162, β = 1378.70)	(32)
Dead	0	—	—	—	—
Discount rate	5%	0%	8%	—	—
Risk adjustment	0.23				(32)

#### Costs of Management of SHPT

The cost and dosage for each drug were obtained from official sources and published literature. Calcitriol was given twice daily, each dose is 0.25 ug. The dose of cinacalcet is 44.7 mg once daily (38). The daily dose of paricalcitol could not be stable until after 18 weeks based on the literature (25). Paricalcitol was administrated by three times a week before meeting the standard, after that altering to once weekly (37). The cost of each drug recorded in detail were summarized in Table 2.

**Table 2:** The costs of SHPT management including paricalcitol, calcitriol, and cinacalcet.

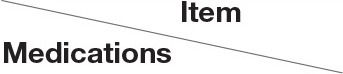	**Cost/tablet (**¥**)**	**Weekly cost (**¥**)**	**Annual cost before up-to-standard (**¥**)**	**Annual cost after up-to-standard (**¥**)**
Paricalcitol	178	534	21766.86	18562.86
Calcitriol	5.4	75.6	3942	3942
Cinacalcet	38	532	27740	27740

#### Costs of Hospitalization

The costs of hospitalization of CKD-5 patients were from a tertiary public hospital, located in Jiangsu Province in China during 2019–2020. Jiangsu province is located on the southeast coast of China. The eastern region like Jiangsu ranks top in the medical resources density index and healthcare services utilization in China in 2016, therefore, it is a good representative. The reason why we choose a single tertiary public hospital is that the few hospitals administrate expensive paricalcitol and cinacalcet, we used the data to show Chinese practical clinical setting by cost. The inclusion criteria of CKD-5D patients are as follows: (1) CKD-5D patients diagnosed with SHPT; (2) Eighteen years of age and above; (3) CKD-5D patients are accompanied by other common complications; The exclusion criteria are as follows: (1) Cross-use of paricalcitol, calcitriol, and cinacalcet during the hospital stay. (2) The patients with other diseases have no association with SHPT, such as depression and tumors.

The annual number of hospitalizations was derived from a US-setting study (28). The average annual number of hospitalizations of paricalcitol, calcitriol was 2.40, 2.61, respectively. Here, we assume that Cinacalcet was consistent with calcitriol, which was a conservative approach toward cinacalcet to perform an analysis in this study due to unavailable data.

#### Costs of Management of ESRD

The costs of transplantation of CKD-5 patients were from a tertiary public hospital, located in Jiangsu Province in China during 2019–2020. The direct medical cost involved kidney source fees, bed charges, nursing care fees, operating expenses, laboratory fees, inspection fees, drug fees, and other fees (material fees) in the first year of receiving kidney transplantation. The medical costs during next year focused on following-up visit costs, including drug fees, laboratory fees, inspection fees, and other fees (material fees).

### Transition Probabilities Including Mortalities

The majority of transition probabilities between health state was from an Italian study (31), partly corrected by the local proportion of HD and PD patients in China (18). In details, the annual probabilities change from kidney transplantation to dialysis was 5.00% (41), as official data in China reported (18), 91.46% of kidney transportation would choose hemodialysis, others would receive peritoneal dialysis. Therefore, we can calculate that 4.60% of kidney transplantation patients receive hemodialysis and 0.40% of that choose peritoneal dialysis. The transition probabilities were shown in Tables 3, 4.

**Table 3:** Transition probabilities between different states in patients with calcitriol and cinacalcet.

	**HD**	**PD**	**Transplantation**	**Die**
**1 year**				
HD	0.829	0	0.033	0.138
PD	0	0.867	0.033	0.100
Transplantation	0.0460	0.00403	0.920	0.030
>**1 year**				
HD	0.829	0	0.033	0.138
PD	0	0.817	0.033	0.150
Transplantation	0.0460	0.00403	0.934	0.016

**Table 4:** Transition probabilities between different states in patients with paricalcitol.

	**HD**	**PD**	**Transplantation**	**Die**
**1 year**				
HD	0.8576	0	0.0254	0.117
PD	0	0.8896	0.0254	0.085
Transplantation	0.0354	0.0031	0.9355	0.026
>**1 year**				
HD	0.8576	0	0.0254	0.117
PD	0	0.8466	0.0254	0.128
Transplantation	0.0354	0.0031	0.9475	0.014

The mortalities data were from same literature (31), widely used in the different clinical settings (31–33), that the mortality rate of paricalcitol decreased by 15% (27), compared to those treated with calcitriol. This historical cohort study enrolling a large number patients revealed that paricalcitol have greater survival advantage over calcitriol.

### Utility

The health utility scores of ESRD patients from the Singapore cross-sectional survey (40) were adopted where the Chinese population accounts for over half the proportion. However, we judged that the health utility score of kidney transplantation population from the UK setting (32) could be applied to the Chinese kidney transplantation population due to unavailable data. Those are measured based on EQ-5D. Health outputs were expressed in terms of quality-adjusted life years (QALYs), which can assess intervention strategy in terms of life time and life quality to reflect the health state of subjects better.

### Base Case Analysis

Base case analysis results were expressed as incremental cost-effectiveness ratio (ICER), which was calculated as incremental costs/incremental QALYs. Based on the World Health Organization (WHO) recommendations, if the ICER < per capita Pross Domestic Product (GDP), the treatment strategy is totally cost effective; if the per capita GDP < ICER < three times per capita GDP, the therapy strategy is acceptable in terms of economic evaluation; if the ICER > three times per capita GDP, we can consider that the therapy strategy is not cost effective. In this study, we assumed that the paricalcitol regimen was cost-effective when the ICER was less than three times per capita gross domestic product (GDP). According to the latest data from the 2020 National Economic and Social Development Statistical Bulletins, the per capita GDP was 7.2447 yuan (42).

### Sensitivity Analysis

The one-way sensitivity analysis and probabilistic simulation analysis were conducted to ensure the robustness of the results. One-way sensitivity analysis was performed by changing a parameter every time to highest or lowest values. A Tornado graph was used to shows the influential variables—the results of one-way sensitivity. Due to unavailable 95% confidence interval (CI), the inpatient expenditures were set to fluctuate between −20 and +20%, the range of utility started from −10 to +10%. Of note, we assume that the price of medicines and devices (including transplantation costs and dialysis costs) involved in disease progression exhibited a mean decrease of 52% because the centralized tender system of medicines and medical devices is being established in China (43).

Probabilistic simulation analysis was conducted by varying all parameters within set different distributions simultaneously in 1000 Monte Carlo simulation iterations to illustrate the results of uncertain analysis and build a cost-effectiveness acceptability curve. In this study, we set costs, utility followed gamma distribution (31, 33), beta distribution, respectively. The value of willingness-to-pay was defined as three times GDP per capita, which was 21.7113 yuan. Here we report that EVPI (expected value of perfect information), interpreted as the average of maximum net monetary benefits, estimates the expected values of uncertainty by combining perfect information with the current known information. We still assume that the benefit patients cohort is 10,000 patients.

## Results

### Base Case Analysis

The results of the base case analysis were summarized in Table 5. Total costs of CKD-5D patients accompanied with SHPT, treated with paricalcitol (472596.007 yuan) paid lower than those with calcitriol and cinacalcet (479521.619 yuan), total utilities as well (2.855 QALYs vs. 2.672 QALYs), in 10 years in the Chinese Healthcare System. Compared to regimen Calcitriol and Cinacalcet, patients who choose Paricalcitol as a regimen need to pay <6925.612 yuan, but gain an increase in Utility (0.183 QALYs). Paricalcitol was dominant as compared to calcitriol and cinacalcet.

**Table 5:** Expected costs and QALYs of two treatment strategies, incremental costs, and incremental QALYs between two treatment strategies, ICERs.

	**Costs (Yuan)**	**Utility (QALYs)**	**ΔC**	**ΔU**
Paricalcitol	472596.007	2.855	−6925.612	0.183
Calcitriol + Cinacalcet	479521.619	2.672		

### Sensitivity Analysis

**Figure 2:**
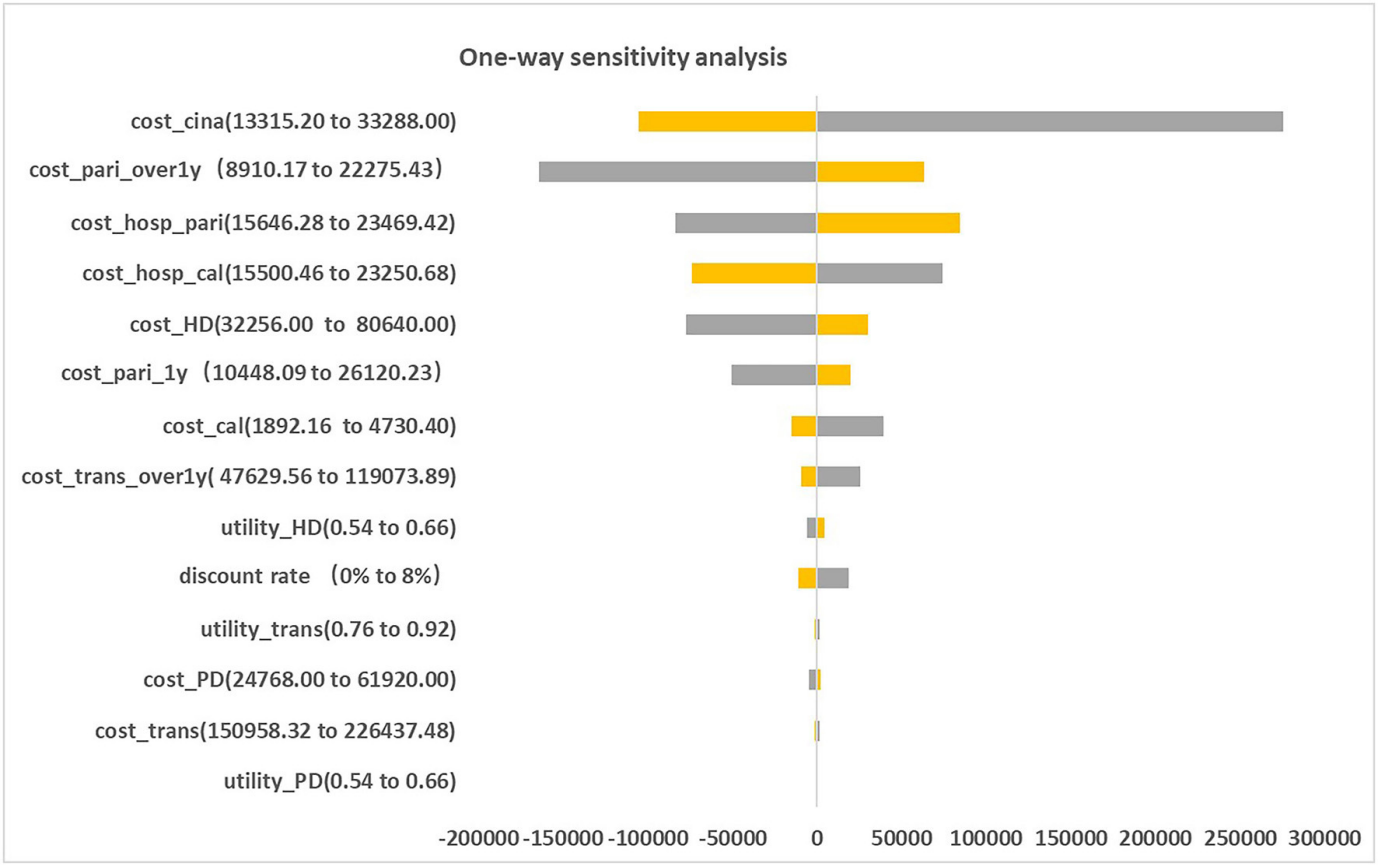
Tornado diagram for two therapies: paricalcitol vs. calcitriol and cinacalcet.

**Figure 3:**
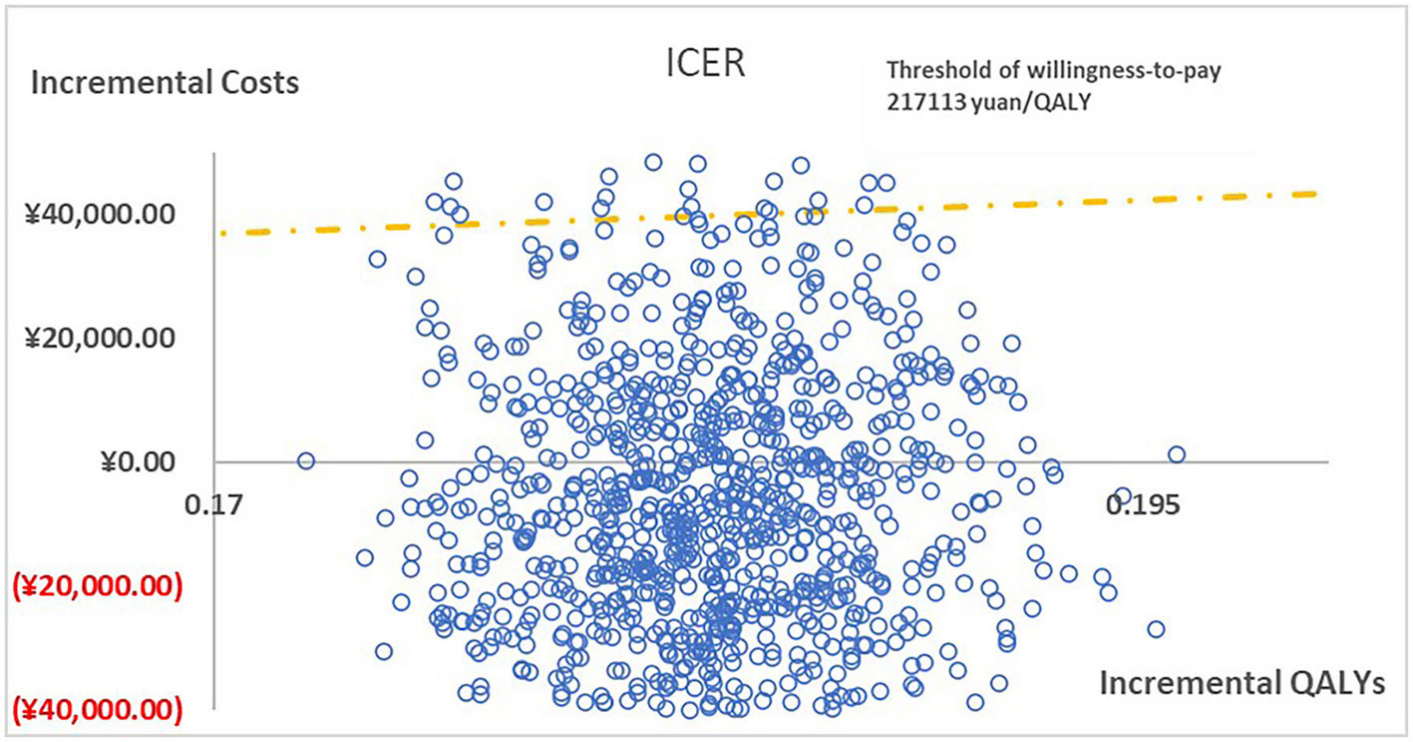
The cost-effectiveness plane illustrated ICERs between paricalcitol and calcitriol + cinacalcet in 1,000 iterations of Monte Carlo simulation.

**Figure 4:**
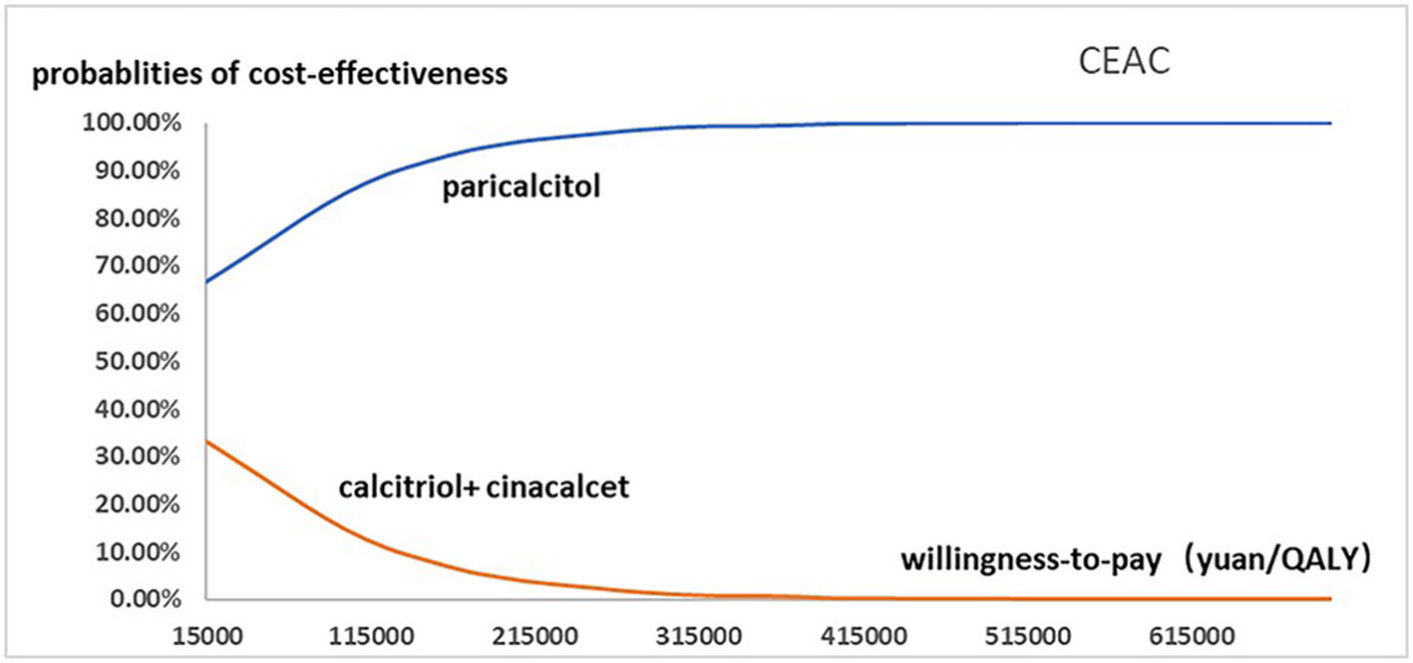
The cost-effectiveness acceptability curve used for probabilistic simulation analysis was drawn to show the probability that paricalcitol vs. calcitriol and cinacalcet was cost-effective at a variety of willingness-to-pay thresholds.

**Figure 5:**
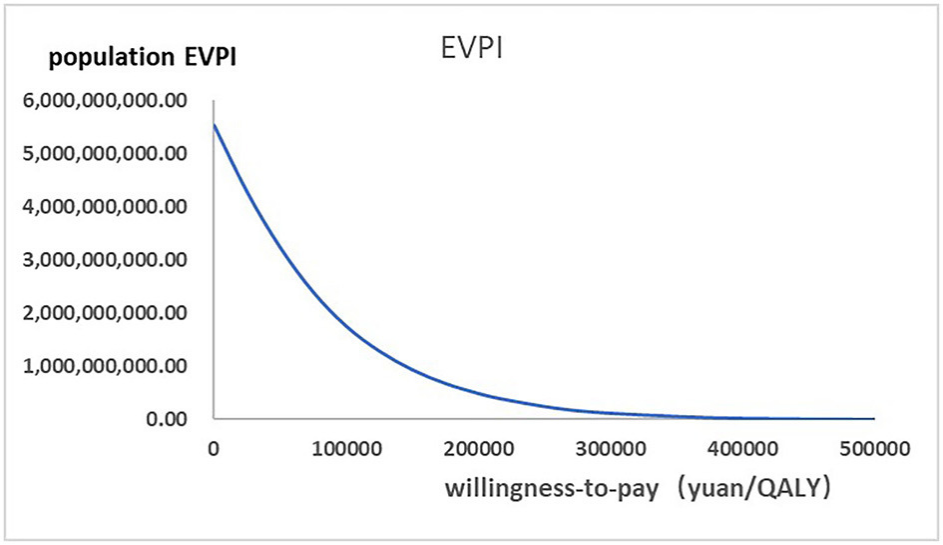
The expected values combining current information and perfect information were shown in the EVPI graph at a variety of willingness-to-pay thresholds.

Figure 2 presented the tornado diagram of one-way sensitivity analysis showing the ICERs in descending order were sensitive to which parameters. In this present study, parameters that might affect ICER. The results showed that this model was relatively robust. The most impactful factor on the total outcome was the price of cinacalcet. The results of probabilistic simulation analysis and were displayed in Figures 3, 4. When willingness-to-pay was 217113 yuan, 96.20% of patients were willing to pay for paricalcitol. Figure 5 displayed the EVPI altering at a variety of willingness-to-pay thresholds.

## Discussion

This is the first study that revealing the cost-effectiveness of paricalcitol in China healthcare system settings. The findings of base case analysis showed that paricalcitol was preferred for CKD-5D patients accompanied with SHPT in China compared to the combination use of calcitriol and cinacalcet, with ICER was negative. This study shows that CKD-5 individuals with SHPT administrated by paricalcitol are an optimal strategy for CKD patients with SHPT in China. The one-way sensitivity analysis suggested that the price of cinacalcet was the most influential factor, the probabilistic sensitivity analysis showed that the probability of paricalcitol was dominant is 96.20% when the threshold of willingness-to-pay was 217113 yuan. Our findings are in line with the previous results done in CKD patients with SHPT from other national studies.

On the other hand, the one-way sensitivity analysis results revealed our model was relatively robust. The main reason that led to this phenomenon was that the magnitudes of decrease in costs of cinacalcet in the model were large (52%). Furthermore, we found it particularly depended on the dosage of paricalcitol after achieving the therapeutic range.

It has been reported that paricalcitol could extend survival and decrease the number of hospitalizations (26, 28). Other advantages have been gradually recognized. For renal transplant recipients, a clinical trial reveals that paricalcitol could decrease proteinuria, which could reduce the risk of kidney transplantation failure (44). Paricalcitol currently is approved to be used in adults, while clinical trials had proven to be effective for children regardless of oral and intravenous (45, 46), besides this, some studies showed that paricalcitol could potentially treat cardiac dysfunction and diabetic nephropathy (47, 48), which suggests the scope of the applicable population should be extended. Furthermore, patients preferred to receive the injection of paricalcitol in the process of getting dialysis due to convenience (11), compared to taking oral calcitriol every day, which can reduce the probability of forgetting to take drugs and increase medication adherence to better control SHPT (49).

With an increase in time of dialysis, vitamin D therapy may be ineffective, parathyroidectomy (PTX) could be considered (50), contributing to improving quality of life (51) and reducing all-cause mortality in ESRD patients with SHPT (52). There are few studies to discuss the cost-effectiveness of PTX, usually compared with cinacalcet (53, 54), which provided new ideas that the cost-effectiveness of PTX for refractory secondary hyperparathyroidism could be investigated in China. Additionally, here we try to discuss more on cinacalcet and other calcimimetics. Cinacalcet has significantly apparent treatment efficacy for severe SHPT patients, it can be used to decrease PTH before receiving PTX and it is a substitute PTX treatment strategy as well (55). Notably, cinacalcet reduces the risk of undergoing PTX (19), also could obtain favorable outcomes for patients with recurrent SHPT after receiving parathyroidectomy (56). Therefore, cinacalcet, a potential therapeutic agent, should be further assessed. A randomized clinical trial (57) demonstrates a novel calcimimetics called etelcalcetide is not inferior to cinacalcet for moderate and severe SHPT patients, in addition, etelcalcetide is administrated intravenous injection to improve adherence and reduce the probability of gastrointestinal adverse effects. However, etelcalcetide has not been launched in China, which is expected to improve cost-effectiveness of medication therapy soon.

However, there are existing problems in terms of treating SHPT in China. Firstly, only 20% of ESRD patients received RRT in China, especially concentrated in large and medium-sized cities (58), which indicated that there are significant geographic differences in ESRD treatment, besides the large population base of CKD patients with SHPT, which may lead to accelerated conditions due to the lack of timely and adequate treatment. Moreover, ESRD patients with complications might achieve worse outcomes, like productivity loss, the absence of caregivers, and even premature death (9). Based on China Kidney Disease Network 2016 annual data report (18), patients with CKD, particularly with SHPT, are associated with increased risk of morbidity and mortality in cardiovascular disease (49), which causes higher medical costs compared to other complications. However, high healthcare costs can not contribute to ideal therapeutic efficacy. According to this study, governments and the relevant departments should pay close attention to optimize medical resource allocation, to reduce the burden of patients. Besides that, over 80% of ESRD patients choose HD as renal replacement treatment in Asia, which parallel to the global conditions, to achieve maximum outcomes of ESRD patients (59). However, HD has worsened outcomes and higher costs than renal transplantation (59), which prompted us to conduct a cost-effectiveness analysis between HD and renal transplantation to provide optimal clinical decisions, which may update awareness and prescribing habits of doctors without considering kidney donations.

This study has several limitations. First, we did some assumptions far away from the real world. Especially, the dosage of drugs, health utility scores of kidney transplantation, and transition probabilities in different health states were not derived from Chinese populations. We could not obtain available data due to no similar studies had been conducted in China. For example, a study revealed that the 1-year survival rate was higher, while the 5-year survival rate was lower than that we adopted (60), whereas we could not confirm that specific clinical setting of the study so that we could not support that. However, according to the requirements of the International Conference on Coordination for Human Drugs (ICH), paricalcitol was approved by National Medical Products Administration (NMPA) in China without local clinical trials, which could demonstrate that the efficacy and safety of paricalcitol in Chinese populations was as same as those in other countries. Therefore, it is reasonable that we adopted the data from published literature from Italia. Second, merely paricalcitol, calcitriol, and cinacalcet were included in the analysis, a possible future of improvement could also incorporate Erythropoietin-treated anemia (a common complication of CKD) and phosphate binders into the analysis. We should be aware of the importance of phosphate control which is significant to mortality and treatment outcomes, moreover, calcium-containing phosphate binders were no longer recommended by China guideline (12) to prevent vascular and soft tissue calcification. Third, we accounted for direct costs using a healthcare perspective, we did not include indirect costs (like productivity loss due to ESRD patients are usually all unproductive, it will be offset from the perspective of the healthcare system and transportation costs) and other costs such as the costs of treating adverse effects. The reason why we did not take adverse effects costs into account is that the adverse effect of paricalcitol, calcitriol and cinacalcet are usually managed by adjusting dosages, which needs real-world data to calculate, we have sufficient data to calculate, which need further exploration.

## Conclusion

The results demonstrated that paricalcitol used for the treatment of secondary hyperparathyroidism in Chronic Kidney Disease when compared to calcitriol and cinacalcet, would be cost-effective from the perspective of the healthcare system in China over 10 years.

## Data Availability Statement

The original contributions presented in the study are included in the article/supplementary material, further inquiries can be directed to the corresponding author.

## Author Contributions

ZZ, LC, and XL designed the whole study. ZZ, HW, LC, and XX were responsible for collecting and analyzing data. ZZ, LC, WF, XW, and XH contributed to preparing the original draft. ZZ, XX, and XL took the responsibility of review and editing. All authors have read and agreed to the published version of the manuscript.

## Conflict of Interest

The authors declare that the research was conducted in the absence of any commercial or financial relationships that could be construed as a potential conflict of interest.
